# 613 Analysis of Cost, Patient Charges and Reimbursement at a Regional Burn Center

**DOI:** 10.1093/jbcr/iraf019.242

**Published:** 2025-04-01

**Authors:** Kaitlyn Malek, Tony Zhao, Richard Lou, Deepak Ozhathil, Anjay Khandelwal

**Affiliations:** Northeast Ohio Medical University; Northeast Ohio Medical University; Akron Children’s Hospital; Akron Children’s Hospital; Adult and Pediatric Burn Institute at Akron Children’s

## Abstract

**Introduction:**

Burn injuries impact close to a half-million people annually in the U.S. and creates a major financial burden. To date, no study has been able to assess the true costs of care have only estimated based on cost:charge ratio, economic modeling or theoretical patient charges. This study utilized novel financial software to analyze actual patient costs, charges and reimbursement, and an assessment of factors influencing cost.

**Methods:**

A retrospective review of adult and pediatric burn patients from 2022-2023 was conducted as the financial software was available starting in 2022. Patient demographics including age, sex, %TBSA burn, etiology, surgical procedures, ventilator days and length of stay (LOS) were collected. Univariate quantile regression and multivariable quantile regression assessed associations between total direct costs (fixed and variable) and clinical factors. Patient charges, all-patient refined diagnosis related group (APR-DRG), payor-mix and reimbursement were calculated as well.

**Results:**

The cohort had a mean age of 40.6 years (range: 0.1–93) and a mean TBSA of 6.83% (range: 0.01–98.4%). Mean LOS was 8.64 days (range: 1–140), with ventilator use averaging 0.68 days (range: 0–136). Payor mix was 44% Medicaid, 22% Medicare and 25% Commercial. Most common APR-DRG was 842 – full thickness burns with skin graft – accounting for 48% of patients.

Mean fixed direct cost was $21,970.74 (range: $27.62–$812,146.10), variable direct cost was $29,799.54 (range: $180.29–$1,358,482.00), with total cost averaging $51,770.28 (range: $296.45–$2,170,628.00), and a mean total cost per day of $8,844.80 (range: $19.76–$839,626.30). Fixed salaries and wages accounted for 29% of the cost and variable supplies accounted for 23%. Mean charges were $201,177 (range: $1,266-$7,142,189) per patient. Total costs represented 27% of total patient charges.

Univariate analysis showed that increased age was associated with lower costs, while TBSA, LOS, days on ventilator, and number of procedures were associated with increased costs. Flame injuries were associated with higher costs and scald injuries were linked to lower costs.

Multivariate analysis which accounted for all covariates considered together, showed age was not a significant predictor but TBSA, LOS, days on ventilator, and the number of procedures remained significant predictors of increased costs.

**Conclusions:**

Etiologically, flame burns are associated with higher costs of burn care. Percent TBSA burn, LOS, days on ventilation, and the number of procedures were the most significant predictors of total cost. The average cost per day for burn patients is just under $9,000/day.

**Applicability of Research to Practice:**

Understanding the real costs of treating burns is crucial for improving resource allocation, identifying cost-effective treatments, and enhancing burn care efficiency. This is the first study to examine the actual cost of burn care in a real-world setting.

**Funding for the Study:**

N/A

